# First-Line Interactive Wound Dressing Update: A Comprehensive Review of the Evidence

**DOI:** 10.3389/fphar.2020.00155

**Published:** 2020-02-28

**Authors:** Carolina D. Weller, Victoria Team, Geoffrey Sussman

**Affiliations:** ^1^ Monash Nursing and Midwifery, Faculty of Medicine, Nursing and Health Sciences, Monash University, Melbourne, VIC, Australia; ^2^ Faculty of Medicine, Nursing and Health Sciences, Monash University, Melbourne, VIC, Australia

**Keywords:** first-line interactive/bioactive dressings, semipermeable film dressings, foam dressings, alginate dressings, hydrofiber dressings, hydrocolloid dressings, hydrogel dressings, hydroactive (foam-like) dressings

## Abstract

Wound management is a significant and growing issue worldwide. Knowledge of dressing products and clinical expertise in dressing selection are two major components in holistic wound management to ensure evidence-based wound care. With expanding global market of dressing products, there is need to update clinician knowledge of dressing properties in wound care. Optimal wound management depends on accurate patient assessment, wound diagnosis, clinicians’ knowledge of the wound healing process and properties of wound dressings. We conducted a comprehensive review of the physical properties of wound dressing products, including the advantages and disadvantages, indications and contraindications and effectiveness of first-line interactive/bioactive dressing groups commonly used in clinical practice. These include semipermeable films, foams, hydroactives, alginates, hydrofibers, hydrocolloids, and hydrogels. In making decisions regarding dressing product selection, clinicians need to ensure a holistic assessment of patient and wound etiology, and understand dressing properties when making clinical decisions using wound management guidelines to ensure optimal patient outcomes. This review has highlighted there is lack of high quality evidence and the need for future well designed trials.

## Introduction

Chronic wounds are associated with a significant health-related quality of life burden and carry high economic costs to society in high income countries (Graves and Zheng, 2014; Guest et al., 2017; Cheng et al., 2018; Olsson et al., 2019). Prevalence is projected to increase due to an aging population and increasing incidence of obesity, diabetes, and cardiovascular diseases (Cheng et al., 2018). Wound management is a significant clinical issue and is a growing economic burden across the globe (Weller and Evans, 2014; Kapp and Santamaria, 2015; Norman et al., 2015; Gray et al., 2018; Pacella et al., 2018). According to the latest Global Wound Care Market 2016 report (Orbis Research, 2017), the Wound Care Market accounted a value of $18.22 billion. This value is projected to reach $26.24 billion by the end of 2023. Optimal wound care requires clinicians’ understanding of wound etiology, wound chronicity, the mechanism and biology of wound healing, and factors that affect wound healing (Ather et al., 2019). The first consideration must be given to accurate diagnosis of wound etiology and appropriate treatment, followed by appropriate dressing selection (Ather et al., 2019; Olsson et al., 2019). Knowledge of dressing products and their properties and dressing selection skills are two major components in clinical decision making and holistic wound care (Dowsett and von Hallern, 2017). Along with patient preferences, clinical expertise is needed to ensure evidence-based wound care (Weller, 2013; Wieten, 2018). However, evidence-based care is frequently suboptimal (Gray et al., 2018; Pacella et al., 2018). To optimize evidence-based decision making, clinicians are encouraged to assess literature on efficacy and cost-effectiveness of dressing products and eliminate the influence of the dressing production industry (Jones et al., 2017). However, studies report that nursing and medical students, interns, and also nurses and general practitioners have insufficient knowledge and lack skills in wound management assessment and dressing selection (Weller and Evans, 2012; Barker et al., 2013; Lemon et al., 2013; Missen et al., 2016; Adderley and Thompson, 2017; Weller et al., 2018; Welsh, 2018). The need to enhance clinicians’ knowledge of dressing selection and assessment skills in wound management is of great importance globally (Zakrasek et al., 2014; Norman et al., 2015; Franks et al., 2016; Münter, 2016; Ayello et al., 2017; Stuart-Shor et al., 2017; Timmins et al., 2018)

Maintaining a moist wound environment to optimize healing is a well-established evidence-based practice (Han and Ceilley, 2017). When compared with traditional passive dressings used to cover wounds, first-line interactive/bioactive dressings provide a moist, conducive environment to facilitate improved healing (Ather et al., 2019). Interactive dressings alter the wound environment and interact with the wound surface to promote wound healing (Sussman, 2014; Weller and Team, 2019). These dressings are often constructed of three layers. The inner level prevents dressing adherence and subsequent trauma to the wound bed. The middle layer absorbs excess exudate and retains absorbed volume and maintains a moist environment. The outer layer prevents bacterial invasion (Han, 2016). The described properties of interactive dressings optimize wound healing processes. Several reviews about first-line interactive dressings have been published (Dhivya et al., 2015; Vowden and Vowden, 2017; Jones et al., 2017; Jones et al., 2018), although these have been brief overviews without evidence of dressing effectiveness. This comprehensive review includes a rigorous review of the effectiveness of first-line interactive wound dressings.

In this article, we discuss the first-line interactive/bioactive dressing groups commonly used in clinical practice, including the semipermeable films, foams, hydroactives, alginates, hydrofibers, hydrocolloids, and hydrogels. We describe physical properties, forms and products, advantages and disadvantages, and indications and contraindications to their use. Additionally, we discuss the available evidence for effectiveness of these groups. This article provides a summary for clinicians, medical and nursing students and recent graduates, and researchers conducting studies in the field of wound management.

## Methods

We completed a comprehensive literature review, using the Seven Steps to a Comprehensive Literature Review (Onwuegbuzie and Frels, 2015) as our framework, including 1) exploring beliefs and topics; 2) initiating the search; 3) storing and organizing information; 4) selecting and deselecting information; and 5) expanding the search using media, observations, documents, experts, and secondary data (MODES) comprise the Exploratory Phase. Analyzing and synthesizing information comprise the Interpretive Phase. Conveying the information, analysis, and the conclusions and implications to the audience comprises the Communication Phase (Onwuegbuzie and Frels, 2015; Williams, 2018).

We have updated earlier works of Weller, Sussman and Team (Weller and Sussman, 2006; Weller, 2009; Sussman, 2014; Sussman, 2016; Weller and Team, 2019). For this purpose we searched the following electronic databases: CINAHL, MEDLINE, PubMed, and Web of Sciences. Additionally, we searched Cochrane database and the Cochrane Wounds Specialised Register. We also used Google Scholar web search engine. We used various combinations of search terms that included the group of dressings, individual dressing products, wound type, and the key word of interest as our search technique. For example, alginate*, burns*, and precaution*; also, Algisite, pressure ulcer*, and evidence. Using Medical Subject Heading (MeSH) browser (https://meshb.nlm.nih.gov/search), we have identified various search terms, *e.g.* pressure ulcer*, bed sore*, bedsore*, decubitus ulcer*, and pressure sore* and included the latest term, *e.g.* pressure injur*.

We searched the latest evidence and, in most cases, included sources published within the last five years, from 2014 until 2019. However, in the absence of recent information, sources from prior to 2014 have been included. We restricted our search to sources published in English language. Different inclusion criteria were developed for individual parts of this review. In the ‘evidence of effectiveness’ sections of various groups of first-line dressings, we included sources, providing the highest quality evidence, such as systematic reviews and randomized controlled trials. In general description and physical properties sections, we used information extracted from published articles, books and book chapters. Although we aimed to include publications that arose from research designs providing the highest level evidence (Burns et al., 2011), in their absence, lower levels of evidence were also considered, including descriptive studies, case series, case reports, and pilot projects; for example, a pilot project on the use of hydrogel dressings for management of Buruli ulcers (Velding et al., 2016). Following step five of the Exploratory Phase, we expanded our search to other sources, and included information from the online *Wound Care Handbook: The professional’s guide to wound product selection* (Healthcare Ltd, 2019); for example, information related to HydroTac and HydroTac Comfort dressings. The following groups of first-line interactive dressings were considered for inclusion in this comprehensive review: the semipermeable films, foams, hydroactives, alginates, hydrofibers, hydrocolloids, and hydrogels (**Table 1**).

**Table 1:** First-line interactive wound dressings: Summary.^1^

Dressings	Forms and products (examples)	Main properties, advantages, and indication	Contraindications and precautions	Evidence of effectiveness
Semipermeable film dressings	Biofilm, Biooclusive, Hydrofilm, Mepilex Film, OpSite, OpSite Flexifix Gentle and Tegaderm	Main properties and related advantages: easily conform to the patient’s body allow some moisture evaporation reduce pain provide a barrier to external contamination allow inspection without dressing removal. Indication: lightly exuding wounds minor abrasions and lacerations superficial burns postoperative sutured wounds radiation dermatitis prevention of pressure injuries.	not suitable for moderately to highly exuding wounds not used in management of infected wounds may result in maceration to the surrounding skin may damage fragile skin newer silicone version will not damage skin	SR^2^ of 6 RCTs^3^: some evidence of effectiveness in prevention and management of radiation dermatitis (application of Mepilex Lite, Mepitel Film, and silver nylon dressings). SR: no clear difference in the incidence of pressure injuries between polyurethane film and hydrocolloid dressings.
Foam dressings	Allevyn (Adhesive, Cavity, Sheet, Life, Gentle), Cavicare, Curafoam, Lyofoam (Max), Mepilex (Sheet,Lite, Border) Permafoam, Tegafoam, Truefoam	Main properties and related advantages: have either hydrophobic or hydrophilic properties silicone membrane allows passage of exudate used to protect wound and periwound provide thermal insulation. Indication: moderately to heavily exuding wounds superficial and cavity wounds infected ulcers skin tears skin grafts and donor sites pressure ulcers venous ulcers (with compression therapy)	not suitable for management of dry wounds, necrotic wounds, hard eschar and wounds requiring frequent review special care is required for people with fragile skin for nonsilicone types may require a retention product	SR: no clear evidence that foam dressings are more effective than other dressings used for management of diabetic foot ulcers SR: no clear evidence that foam dressings are more effective than other dressings used for management of pressure injuries SR: moderate evidence that there is no significant difference in partial thickness burns healing between silver-containing foam dressing and the traditionally used gauze with silver sulphadiazine. Some benefits of foams in reduced wound pain and decreased infection rates. RCT: use of a soft silicone foam dressing combined with standard preventive care provided a statistically and clinically significant benefit in reducing incidence and severity of hospital acquired pressure injuries compared to standard preventive care. RCT: the use of the Mölnlycke Mepilex Border Sacrum and Mepilex Heel dressings offered a significant additional protective benefit in prevention of pressure injuries in high-risk aged care residents
Hydroactive dressings (foam-like)	Biatain surface sheet, Cutinova Hydro surface sheet thick, PolyMem surface sheet and cavity dressing, and Tielle surface sheet	Main properties and related advantages: absorb exudate quickly and effectively, reducing the risk of maceration provide moisture to dry wounds, facilitating faster healing do not stick to the wound bed, reducing patient trauma on removal soothe painful wounds, providing greater patient comfort and tolerability protect the wound from bacteria, reducing the risk of infection Indication: highly exuding wounds surface and cavity wounds pressure injuries venous leg ulcers minor burns	not indicated for lightly exuding and dry wounds	No RCTs or SRs on hydroactive dressings were have been published to date.
Alginate dressings	Algisite, Algoderm, Curasorb, Kaltostat, Seasorb Alginate, Suprasorb A, Sorbsan, and Tegaderm Alginate are manufactured as a surface sheet and/or a rope	Main properties and related advantages: are highly absorbent form a gel with exudate create a moist environment can be easily removed are hemostatic are flexible can be used to pack wounds. Indication: moderately to severely exuding wounds bleeding sites, cavities donor sites pressure ulcers exuding venous leg ulcers	should not be used on wounds with hardened eschar or dry wounds inflammatory or anaphylactic reactions can occur alginate rope dressings should be used with caution in narrow or very deep sinuses because complete removal may be difficult not recommended in management of wounds with anaerobic infections	SR: Three SRs have been conducted to compare relative effects of alginate dressings with alternative treatments in management of venous leg ulcers, diabetic foot ulcers, and pressure ulcers. The results reported that there is no clear current evidence to suggest that alginate dressings are more or less effective than other types of dressing in wound healing. RCT: Prospective RCT on the use of Algisite M, Cuticerin, and Sorbact as donor site dressings in pediatric split-thickness skin grafts reported there are no significant differences between the three dressings in regard to both primary study outcomes of pain and time to re-epithelialization, and secondary outcomes of scarring, itch, and cost.
Hydrofiber dressings Other fiber dressings:	Aquacel, Aquacel Foam, Aquacel Extra Exufiber, Durafibee Carboflex is a combination of a Calcium Alginate, a Hydrofiber and activated charcoal	Main properties and related advantages: fibers form a gel on contact with exudate highly absorbent vertical wicking of exudate helps to reduce maceration of periwound can be kept in place until the dressing is saturated facilitate autolytic debridement Indication: wounds with moderate to excessive exudate	should not be used on dry wounds as they can produce a fibrinous residue if used in mildly exuding wounds, clinicians may need to soak the dressing in sterile water or saline before removal to reduce trauma	A SR and meta-analysis to identify the optimal wound dressing material following total hip and knee arthroplasty reported wounds managed with hydrofiber dressings were significantly less likely to have wound complications than those managed with passive dressings. Hydrofiber dressings also showed better fluid handling capacity than passive dressings. A SR aimed to determine the effects of different methods on venous leg ulcer debridement reported hydrofiber dressings are less superior than biocellulose in a total mean reduction in ulcer size. Small scale retrospective study showed that the use of hydrofiber Aquacel Extra for skin graft fixation was effective and simple to use in the clinical setting. Retrospective review of medical histories showed that more complex, silver containing hydrofiber dressing Aquacel Ag has been reported to promote faster healing of partial thickness burns in pediatric patients and reduced their hospital stay.
Hydrocolloid dressings	Combiderm, Comfeel, DuoDerm, Hydrocoll, Replicare, Suprasorb H, Tergasorb, Tegaderm Hydrocolloid Ultec	Main properties and related advantages: facilitate angiogenesis and granulation facilitate acidic environment to inhibit bacterial growth promote autolytic debridement adhere well to high friction areas, such as the heels and sacrum reduce pain can be used as primary or secondary dressings Indication: lightly to moderately exuding wounds that would benefit from autolytic debridement partial- and full-thickness acute and chronic wounds. management of pressure injuries as protective dressings to prevent device-related pressure injuries in intubated patients in ICU minor burns and abrasions. leg ulcers, pressure ulcers, burns, and donor sites	should not be used in management of dry and high exudate wounds are not recommended on clinically infected wounds wound tissue assessment is paramount if applied for a long period of time should be discontinued before hypergranulation occurs caution in use in diabetic foot ulcers as hydrocolloids may facilitate growth of anaerobic bacteria.	SRs: there is limited evidence that hydrocolloids are more effective than other dressings, in managing diabetic foot ulcers and pressure injuries. RCT: Findings from an RCT on donor site wound dressings after split-skin grafting report time to complete re-epithelialization with hydrocolloid dressings was shorter when compared with any other dressings.
Hydrogel dressings	Thickest Sterile hydrogels: Purilon and IntraSite Thinnest preserved hydrogels: Solosite and Solugel Sheet hydrogels: Hydrosorb, HydroTac, HydroTac Comfort, Intrasite Conformable, Suprasorb G Sheet Two-layer hydrogels: Oxyzyme and Iodozyme Antimicrobial hydrogels: Flaminal Hydro and Forte	Main properties and related advantages: soothing and cooling effects on the skin facilitates autolytic debridement without damage to epithelial cells or granulation enzymes lactoperoxidase and glucoseoxidase provide the antibacterial action Indication: minimally exuding wounds or dehydrated wounds thicker products are used for protection of exposed tendon and/or bone from dehydrating and rehydrating eschar before debridement thinner products are useful for soothing burns and acute chicken-pox lesions grazes/lacerations donor sites pressure injuries radiation oncology burns.	should not be used in highly exuding wounds provide poor bacterial barrier may contain propylene glycol that may cause allergic reactions in older people the wound may be irrigated with saline solution for easy removal of hydrogel dressings hydrogels can remain in place for no longer than three days.	A SR on hydrogel dressing use in management of pressure ulcers reported there is no clear evidence that hydrogel dressings are more or less effective than other treatments or that different hydrogels have different effects. A SR on hydrogel dressing use in management of diabetic foot ulcers reported low level evidence that hydrogel dressings are more effective in healing lower grade diabetic foot ulcers than basic wound contact dressings. A SR indicated there is limited evidence that superficial and partial thickness burns managed with hydrogel dressings heal more quickly than those managed with usual care. HydroTac was piloted in management of Buruli ulcers and reported that the use of foam dressings without a hydrogel component may be more effective.

^1^We designed this table using multiple sources. These resources have been acknowledged in the reference list.

^2^SR, Systematic review.

^3^RCT, Randomised controlled trial.

## Results

### Semipermeable Film Dressings

#### General Description and Physical Properties

Semipermeable film dressings are permeable to gas and impermeable to bacteria and liquid. These dressings comprise of thin elastic polyurethane films, which are conformable and adhesive. Transparency of films allows wound inspection without dressing removal (Weller and Team, 2019).

#### Forms and Products

Biofilm, Biooclusive, Hydrofilm, Mepilex Film, OpSite, OpSite Flexifix Gentle and Tegaderm are example products (Vowden and Vowden, 2017). OpSite and Tegaderm have similar moisture vapor transmission rate (MVTR) characteristics (Vowden and Vowden, 2017). However, the Island forms OspSite PostOp has been reported to have a higher MVTR (Vowden and Vowden, 2017).

#### Advantages and Disadvantages

Semipermeable film dressings: (i) easily conform to the patient’s body; (ii) allow some moisture evaporation; (iii) reduce pain; (iv) provide a barrier to external contamination; and (v) allow inspection without dressing removal. The main disadvantages are dressings may be traumatic on removal; and excessive pooling of exudate may occur when used on heavily exuding wounds (Arroyo et al., 2015; Vowden and Vowden, 2017).

#### Indications and Method of Use

Island dressings have a central nonstick pad and can absorb slightly more exudate than other films. The frequency of dressing change will depend on the wound location, type and size, and may be left on the wound unchanged for up to seven days. Film dressings are usually used as primary dressings for minor abrasions, lacerations and burns. Films can be used to waterproof a primary dressing, such as foam (Bryant and Nix, 2015). They are also often used as a postoperative layer over sutured wounds to keep them dry (Dabiri et al., 2016). Barrier polymer films, such as foam applicators and spray, are used for protection of periwound skin from moisture-related damage and prevention of dressing adhesion (Vowden and Vowden, 2017). Films can also be used as a protective layer to prevent superficial pressure injuries (Haynes, 2013).

#### Contraindications and Precautions

Island films are not suitable for highly exuding wounds because they are nonabsorbent. They should be discontinued if excessive exudate pools under the dressing (Arroyo et al., 2015; Vowden and Vowden, 2017). Films may result in maceration of the surrounding skin, increasing the risk of infection (Dabiri et al., 2016). Clinicians should be cautious in applying and removing films from fragile skin to avoid skin damage. There are newer silicone versions that are safe. They should not be used in management of infected wounds (Vowden and Vowden, 2017).

#### Evidence of Effectiveness

Semipermeable film dressings were found to be beneficial in the prevention and management of radiation-induced skin reactions, such as radiation dermatitis of different grades from local erythema to moist desquamation, as findings of a systematic review (Fernández-Castro et al., 2017) indicated. This review was based upon findings from six randomized controlled trials (RCTs), which analyzed the application of Mepilex Lite, Mepitel Film and silver nylon dressings in patients with breast cancer, head and neck cancer, and lower gastrointestinal cancer. A systematic review of dressings and topical agents for preventing pressure injuries (Moore and Webster, 2018) reported there was no clear difference in the incidence of pressure injuries between polyurethane film and hydrocolloid dressings.

### Foam Dressings

#### General Description and Physical Properties

Foam dressings are made from polyurethane, and some forms have a coating of soft silicone to allow the dressing to remain in place and be removed without trauma. This silicone membrane allows for the exudate to pass into the insulating foam. Foams can have either hydrophobic or hydrophilic properties (Weller and Team, 2019).

#### Forms and Products

Foam dressing products are available in a wide range of sizes or cavity filling shapes. These products may be adhesive or nonadhesive. Allevyn, Permafoam, Lyofoam Max, Mepilex, Suprasorb P PU are examples of foam dressing products (Dumville et al., 2017).

#### Advantages and Disadvantages

Foams are highly absorbent, protective, insulating and possessing a property that conform to body surfaces. Foam dressings facilitate a moist wound environment required for wound healing and absorb excess exudate, decreasing the risk of skin maceration (Nielsen and Fogh, 2015). Foam dressings do not require frequent changes due to their properties that conform to wound shape, reduce dead space, and absorb large amounts of exudate. They can be left in place for about a week, depending on the level of exudate. However, cavity foam dressings are usually nonadhesive and require the use of secondary dressings to keep them in place, and thus, increasing the cost of wound management (Weller and Team, 2019). One of the disadvantages of foam dressings is the potential for ingrowth of newly formed tissue into the dressing due to infrequent dressing changes, which may result in shearing trauma upon dressing removal (Lee et al., 2016). Another disadvantage is that the set size of the foam product may be limited by the size of the wound.

#### Indications and Method of Use

Foam dressings are generally used as primary dressings, although they may be used as the secondary dressing with alginate or hydrogel dressing (Vowden and Vowden, 2017). They are used in the management of mildly to moderately exuding wounds and are suitable for burns, chronic wounds, deep ulcers, and wound-shape cavities (Jung et al., 2016).

#### Contraindications and Precautions

Foam dressings are not suitable for management of dry wounds, necrotic wounds, hard eschar and wounds requiring frequent review. Special care is required when adhesive materials are used to keep foam dressings fixed in older people, as their skin is characterized by increased fragility and susceptibility to breakdown. Tubular retention bandages or light weight cohesive bandages to fix foam dressing in place provide a safer option in this population group (Idensohn et al., 2019).

#### Evidence of Effectiveness

Two systematic reviews produced no clear evidence that foam dressings are more effective than other dressings used for the management of diabetic foot ulcers (Dumville et al., 2013a) and pressure injuries (Walker et al., 2017). In the absence of robust evidence, clinicians are advised to take into account the wound management properties by each dressing type and to evaluate the patient, the wound, and care context when making decisions to use foam dressings (Walker et al., 2017). A randomized controlled trial conducted in the USA reported that the use of a soft silicone foam dressing combined with standard preventive care provided a statistically and clinically significant benefit in reducing incidence and severity of hospital acquired pressure injuries (HAPIs) in intensive care patients when compared to patients who received standard preventive care (Kalowes et al., 2016). A prospective dual-center sham study conducted in Japan that involved patients undergoing elective spinal surgery reported that soft silicone foam dressing was more effective than polyurethane film dressing for the prevention of intraoperatively acquired pressure injuries (IAPIs) (Yoshimura et al., 2018). A randomized controlled trial of clinical effectiveness of multi-layer silicone foam dressings used for the prevention of pressure injuries in high-risk aged care residents in Australia reported that the use of the Mölnlycke Mepilex Border Sacrum and Mepilex Heel dressings offered a significant additional protective benefit to the participants in this population group (Santamaria et al., 2018). A systematic review (Chaganti et al., 2019) of foam dressings for the management of partial thickness burns reported there is moderate evidence that there is no significant difference in burn healing between silver-containing foam dressing and the traditionally used gauze with silver sulphadiazine. However, foam dressings were reported to have the benefits of reduced wound pain and decreased infection rates (Chaganti et al., 2019).

### Hydroactive Dressings (Foam-Like)

#### General Description and Physical Properties

Hydroactive dressings are multilayered polymer dressings. They are highly absorbent; some have a waterproof outer layer and a surface adhesive. Although hydroactive dressings are similar to foams, they have a different action for absorbing exudate. Hydroactive dressings draw fluid into the structure of the polymer and trap the exudate to maintain a moist environment; whereas, foams absorb exudate by a syphon effect (de Vries, 2018).

#### Forms and Products

Hydroactive dressings combine a net shaped hydrogel wound contact layer and absorbent foam. Biatain surface sheet, Cutinova Hydro surface sheet thick, PolyMem surface sheet and cavity dressing, and Tielle surface sheet are examples of hydroactive dressings (de Vries, 2018).

#### Advantages and Disadvantages

The main advantages of the hydroactive dressing are that they: i) absorb exudate quickly and effectively, reducing the risk of maceration; (ii) provide moisture to dry wounds, facilitating faster healing; (iii) do not stick to the wound bed, reducing patient trauma on removal; (iv) soothe painful wounds, providing greater patient comfort and tolerability; (v) protect the wound from bacteria, reducing the risk of infection (de Vries, 2018).

#### Indications and Method of Use

Hydroactive dressings are indicated for highly exuding wound surface and cavity wounds, including pressure injuries, venous leg ulcers, and minor burns (Augustin et al., 2016). Due to the ability to contract and expand without causing constriction, they are particularly useful over joints (de Vries, 2018).

#### Contraindications and Precautions

Hydroactive dressings are not indicated for lightly exuding and dry wounds (de Vries, 2018).

#### Evidence of Effectiveness

No randomized controlled trials or systematic reviews on hydroactive dressings have been published to date. Low quality small scale, noncontrolled experimental study reported efficient use of occlusive foam dressing TenderWet-plus^®^–Hartmann impregnated with polyhexamethylene biguanide in debridement and bacterial load management of infected wounds of mixed etiology (Mancini et al., 2018).

### Alginate Dressings

#### General Description and Physical Properties

Alginates may be either calcium or calcium sodium salts of alginic acid. When applied to a wound, the calcium in the alginate reacts with the sodium salts present in the wound exchanges, forming sodium alginate, which is a hydrophilic gel (Lee and Mooney, 2012). Alginate is a naturally occurring biopolymer that is sourced from brown algae (*Phaeophyceae)*, including *Ascophyllum nodosum, Laminaria hyperborea, Laminaria japonica, Macrocystis pyrifera,* and *Laminaria digitate* (Aderibigbe and Buyana, 2018). Soluble sodium alginate gel forms as a result of an active ion exchange of calcium ions for sodium ions at the wound surface, resulting in absorption of fluid at the wound site (Aderibigbe and Buyana, 2018).

#### Forms and Products

Alginates are processed to form a number of dressing types that vary in their shape and size. They can be adapted to form wafers, nanofibers and topical formulations (Aderibigbe and Buyana, 2018). Alginate dressings are available as freeze-dried porous sheets or flexible fiber dressings. They can absorb up to 20 times their weight in fluid, and are easy to remove from the wound (O'Meara et al., 2015). Algisite, Algoderm, Curasorb, Kaltostat, Seasorb Alginate, Suprasorb A, Sorbsan, and Tegaderm Alginate are manufactured as a surface sheet and/or a rope (Aderibigbe and Buyana, 2018).

#### Advantages and Disadvantages

The main advantages of the alginate dressings are that they: (i) are highly absorbent; (ii) form a gel with exudate; (iii) create a moist environment; (iv) can be easily removed; (v) are hemostatic; and (vi) are flexible and can be used to pack wounds (Hickman et al., 2018; Lee and Mooney, 2012). The main disadvantages are that they are nonadhesive, requiring a secondary dressing (Weller and Team, 2019) and there have been reports of allergic reactions (McCarthy et al., 2018) when there is not enough moisture within the wound cavity to form a removable gel.

#### Indications and Method of Use

Alginate dressings are highly absorbent, biodegradable dressings used in moderately to severely exuding wounds. One of the benefits of using an alginate dressing is their efficacy in absorbing excess moisture from wounds (Weller and Team, 2019). They are used on bleeding sites, exuding leg ulcers, cavities and donor sites (Hickman et al., 2018; Dumville et al., 2013d; Dumville et al., 2015a; McBride et al., 2018). Some calcium alginate dressings facilitate hemostasis in bleeding wounds due to the active release of calcium ions that aid the clotting mechanism. Alginates require a secondary dressing, such as hydrocolloids or foams, to keep the alginate dressing in place and prevent it from drying out (Vowden and Vowden, 2017). In general, alginates can be kept in place from one to three days depending on the level of exudate, and they are changed when they have fully converted to a gel. When used on a donor site, they can remain in place for up to seven days. Alginate dressing is often placed over the donor area after skin harvesting and covered with a foam or a film dressing to keep it in place (McBride et al., 2018).

#### Contraindications and Precautions

As alginates are highly absorbent; they should not be used on wounds with hardened eschar or dry wounds (Vowden and Vowden, 2017). Inflammatory or anaphylactic reactions can occur in the use of alginate dressings, and therefore clinicians are asked to use caution when using these dressings (McCarthy et al., 2018). Alginate rope dressings should also be used with caution in narrow or very deep sinuses because complete removal may be difficult. Clinicians should avoid packing the material tightly into the wound space (Vowden and Vowden, 2017). Alginate dressings are not recommended in the management of wounds with anaerobic infections. If applied on infected wound, clinicians should ensure that the secondary dressing is nonocclusive (Vowden and Vowden, 2017).

#### Evidence of Effectiveness

Three systematic reviews have been conducted to compare relative effects of alginate dressings with alternative treatments in management of venous leg ulcers (O'Meara et al., 2015), diabetic foot ulcers (Dumville et al., 2013d), and pressure ulcers (Dumville et al., 2015a). The results reported that there is no clear current evidence to suggest that alginate dressings are more or less effective than other types of dressing in wound healing. The available RCTs included in these systematic reviews were considered to be of low or unclear methodological quality. Recent prospective randomized controlled trial on the use of Algisite M, Cuticerin, and Sorbact as donor site dressings in pediatric split-thickness skin grafts conducted in Australia reported there are no significant differences between the three dressings in regard to both primary study outcomes of pain and time to re-epithelialization, and secondary outcomes of scarring, itch, and cost (McBride et al., 2018).

### Hydrofiber and Other Fiber Dressings

#### General Description and Physical Properties

Hydrofiber dressings are nonwoven sodium carboxymethyl cellulose spun into fibers (Vowden and Vowden, 2017). Hydrofiber dressings are a fiber rope or dressing that forms a firm gel when in contact with fluid and thus have some of the properties of alginates (Dabiri et al., 2016). They can absorb up to 25 times their own weight in fluid (Sood et al., 2014).

#### Forms and Products

Aquacel is an example of a hydrofiber dressing. Aquacel fibers form a gel on contact with exudate, which helps to maintain a moist wound environment (Tickle, 2012). The vertical wicking of exudate helps to reduce maceration of periwound. Aquacel Foam and Aquacel Extra are other examples of this product. These dressings are more absorbent than alginates and promote nontraumatic dressing removal (Tickle, 2012). There are other fiber dressings, polymer fibers that absorb exudate and are used in cavity wounds, *e.g.* Exufiber, Durafibee (Beldon, 2016). Carboflex is a combination of a Calcium Alginate, a Hydrofiber and activated charcoal which is useful in exuding malodorous wounds (Sood et al., 2014; Dabiri et al., 2016).

#### Advantages and Disadvantages

The main advantages of the hydrofiber dressings are that they are highly absorbent and have no lateral wicking, which protects periwound (Beldon, 2016; Dabiri et al., 2016). They also facilitate autolytic debridement (Dabiri et al., 2016). Hydrofiber dressings are nonadherent and require secondary dressings to keep them in place.

#### Indications and Method of Use

Hydrofiber dressings are used in the management of wounds with moderate to excessive exudate. They can be kept in place until the dressing is saturated (Dabiri et al., 2016).

#### Contraindications and Precautions

Hydrofiber dressings should not be used on dry wounds as they can produce a fibrinous residue on the wound bed surface. This residue should be gently rinsed away at dressing changes. If used in mildly exuding wounds, clinicians may need to soak the dressing in sterile water or saline before removal to minimize wound bed trauma and reduce associated pain (Dabiri et al., 2016; Beldon, 2016).

#### Evidence of Effectiveness

There is evidence that hydrofiber dressings are more effective than passive dressings in the management of postsurgical wounds. Findings from a systematic review and meta-analysis to identify the optimal wound dressing material following total hip and knee arthroplasty reported that wounds managed with hydrofiber dressings (OR, 0.28; 95% CI, 0.20–0.40) were significantly less likely to have wound complications than those managed with passive dressings (Sharma et al., 2017). Hydrofiber dressings also showed better fluid handling capacity than passive dressings in terms of number of patients requiring early dressing change and mean number of dressing changes. However, there is insufficient evidence to conclude whether the use of hydrofiber dressings reduces periprosthetic joint infection (Sharma et al., 2017). A systematic review aimed to determine the effects of different methods on venous leg ulcer debridement reported hydrofiber dressings are less superior than biocellulose in a total mean reduction in ulcer size (Gethin et al., 2015). The results of a retrospective study showed that the use of hydrofiber Aquacel Extra for skin graft fixation was effective and technically very simple to use in the clinical setting (Yen et al., 2018). More complex dressing, such as silver containing hydrofiber dressing Aquacel Ag, has been reported to promote healing of partial thickness burns in pediatric patients (Lau et al., 2016).

### Hydrocolloid Dressings

#### General Description and Physical Properties

Hydrocolloids are moisture-retentive dressings, containing gel-forming agents, such as gelatin, sodium carboxymethylcellulose, and pectin. In the presence of wound exudate, hydrocolloids absorb liquid, forming a gel, which helps to maintain moist environment (de Vries, 2018).

#### Forms and Products

Many hydrocolloid dressings combine the gel-forming properties with elastomers and adhesives which are usually applied to a carrier, such as film or foam to form an absorbent, waterproof, self-adhesive wafer (de Vries, 2018). In sheet form, the polymer outer layer can be either occlusive or semiocclusive. Hydrocolloid interaction facilitates autolytic debridement. This property helps to reduce dressing frequency to up to one-week wear time, depending on the type of hydrocolloid dressing and the amount of exudate. Hydrocolloids are also available in powders and paste, which increase exudate absorption and decrease dead space in the wound cavity (Weller and Team, 2019). Comfeel, DuoDerm, Hydrocoll, Suprasorb H, and Tegaderm Hydrocolloid are examples of hydrocolloid dressings (de Vries, 2018).

#### Advantages and Disadvantages

The main advantages of the hydrocolloid dressings are moisture retention and pain free removal. The occlusive properties provide a good barrier to water, oxygen, or bacteria; however, there is a potential for anaerobic bacteria to grow in a hypoxic environment. These properties help facilitate angiogenesis and granulation. Additionally, hydrocolloid dressings decrease wound surface pH of the facilitating acidic environment to inhibit bacterial growth (Vowden and Vowden, 2017).

#### Indications and Method of Use

Hydrocolloid dressings are suitable for partial- and full-thickness acute and chronic wounds. They promote autolytic debridement with sloughy or necrotic tissue (Vowden and Vowden, 2017). They adhere well to high friction areas, such as heels and the sacrum and are used in the management of pressure injuries (Keogh et al., 2013). Hydrocolloid dressings are also used as protective dressings to prevent device-related pressure injuries in intubated patients in the ICU (Garrubba, 2017). They are also used in the management of minor burns and abrasions.

#### Contraindications and Precautions

Health professionals should avoid the use of hydrocolloid dressings in managing dry and high exudate wounds. Due to the semiocclusive nature, hydrocolloids with a waterproof backing are not recommended on clinically infected wounds (de Vries, 2018). Hypergranulation may occur with prolonged use of hydrocolloids in moderately to highly exuding wounds. When applying hydrocolloids for long periods, regular wound tissue assessment is paramount to ensure that hydrocolloid dressings are discontinued before hypergranulation occurs (Swanson, 2014). Some studies caution use in diabetic foot ulcers (Lithner, 1990; Lawrence, 1995; Foster et al., 1997) as hydrocolloids may facilitate growth of anaerobic bacteria (de Vries, 2018).

#### Evidence of Effectiveness

Hydrocolloid dressings are used as primary or secondary dressings for the management of pressure ulcers, chronic venous ulcers, diabetic foot ulcers, burns, partial thickness wounds, and split-thickness skin graft donor site wounds with various degrees of effectiveness (de Vries, 2018; Brown and Holloway, 2018). However, there is limited evidence that they are more effective than other dressings, particularly in managing diabetic foot ulcers (Dumville et al., 2013b) or pressure ulcers (Pott et al., 2014). The results of a cost comparison study of hydrocolloid dressing *versus* transparent polyurethane film in pressure ulcer prevention reported that the mean cost per dressing change per participant was lower when using the transparent polyurethane film than when using the hydrocolloid dressing (Dutra et al., 2016). Findings from a randomized controlled study of donor-site wound dressings after split-skin grafting (Brölmann et al., 2013) reported that time to complete re-epithelialization with hydrocolloid dressings was improved by seven days when compared with any other dressings.

### Hydrogel Dressings

#### General Description and Physical Properties

Hydrogels are composed of complex hydrophyllic polymers with a high (90%) water content. They are water-insoluble polymers that expand in water. Hydrogel dressings are semiocclusive. They are used to hydrate wounds, rehydrate eschar, and aid autolytic debridement (Weller and Team, 2019).

#### Forms and Products

Hydrogel dressings are available in amorphous gel, sheet or sheet hydrogel-impregnated dressings. Hydrogel viscosity varies. Purilon and IntraSite are two of the thickest sterile gels. This property helps them stay in the cavity of the wound. Solosite and Solugel are two of the thinnest hydrogels, which are easily spread over a large area; however, they both contain chemical preservatives (Weller and Team, 2019). Hydrogels containing antimicrobial agents, antibiotics, and hyaluronic acid have been developed (Vowden and Vowden, 2017). Antimicrobial hydrogels, *e.g.* Flaminal Hydro and Forte, incorporate two enzymes, lactoperoxidase and glucose oxidase, and inhibit bacterial growth (Beele et al., 2012; White, 2014; Finnegan and Percival, 2014). Oxyzyme and Iodozyme are examples of two-layer hydrogel dressings which release both oxygen and iodine onto the wound surface (Vowden and Vowden, 2017). Hydrosorb, HydroTac, HydroTac Comfort, Intrasite conformable, and Suprasorb G Sheet are examples of sheet hydrogels (Healthcare Ltd, 2019).

#### Advantages and Disadvantages

Hydrogel dressings provide a moist environment that facilitates cell migration and absorbs some exudate. Autolytic debridement without damage to epithelial cells or granulation is another advantage of hydrogel dressings (Kumar et al., 2017). The main disadvantage of hydrogel dressings is that they provide poor bacterial barrier.

#### Indications and Method of Use

Hydrogel dressings are suitable for the management of wounds with minimal to moderate exudate. Amorphous hydrogels can be applied generously onto a wound and covered with a secondary dressing, such as film or foam. Hydrogels can remain in place for no longer than three days. Hydrogel products can be used in the management of pressure ulcers, skin tears, and surgical wounds. They have marked soothing and cooling effects on the skin, which is particularly valuable in management of burns, including radiation oncology burns, and painful wounds (Vowden and Vowden, 2017). They are safe on neonatal skin. In addition to their use in wound care, thin hydrogels are useful in the management of chicken pox lesions and shingles (Lau, 2015). HydroTac and HydroTac Comfort are used in the management of wounds in the granulation and epithelialization stages of healing. HydroTac facilitates sufficient absorption of exudate with top film water vapor permeability, which provides an optimal moist wound environment required for effective wound healing. It is indicated in the management of slightly to moderately exuding wounds (Healthcare Ltd, 2019). HydroTac Comfort has the same advantages to the wound bed but is produced with an additional acrylic adhesive border, which provides an all-in-one dressing for waterproof protection of the wound surface (Healthcare Ltd, 2019).

#### Contraindications and Precautions

Health professionals should be aware that some amorphous gels may contain propylene glycol that may cause allergic reactions in older people. For easy removal of hydrogel dressings, the wound may be irrigated with saline solution (Weller and Team, 2019).

#### Evidence of Effectiveness

The results of a systematic review on hydrogel dressing use in the management of pressure ulcers reported that there is no clear evidence that hydrogel dressings are more or less effective than other treatments or that different hydrogels have different effects (Dumville et al., 2015b). Most trials included in this review were very small and inadequately reported, increasing the risk of bias. A systematic review on hydrogel dressing use in the management of diabetic foot ulcers reported there is low level evidence that hydrogel dressings are more effective in healing lower grade diabetic foot ulcers than basic wound contact dressings (Dumville et al., 2013c). However, there is risk of bias in the original studies, making this finding uncertain. There is limited evidence that superficial and partial thickness burns managed with hydrogel dressings heal more quickly than those managed with usual care (Wasiak et al., 2013). The studies included in this systematic review were small trials of poor quality and high risk of bias (Wasiak et al., 2013). HydroTac was piloted in the management of Buruli ulcers and reported the use of foam dressings without a hydrogel component may be more effective (Velding et al., 2016).

### Discussion

Although the development of advanced wound dressings is on the rise, and many new-age products have entered the wound market (Ahangar et al., 2018), first-line interactive/bioactive dressings are most commonly used in clinical practice. We have described the physical properties of the main groups of the first-line interactive/bioactive dressings, provided forms and examples of these products, discussed their advantages and disadvantages, as well as the indication and contraindications. We have also provided the latest available evidence of effectiveness. Despite facilitating evidence-based practice in wound care over the last two decades, to date the evidence of effectiveness of the included groups of first-line interactive/bioactive dressings is limited. (Game and Jeffcoate, 2016; Pagnamenta, 2017; Team et al., 2019). The need for high quality randomized controlled trials on the effectiveness of dressing products with further evidence synthesis, including systematic reviews, overviews of reviews and network meta-analysis, are warranted (Dumville et al., 2013c; Wu et al., 2015; Moore and Webster, 2018; Norman et al., 2018). However, there are many challenges related to evidence generation in wound care (Team and Weller, 2019), including dressing selection (Pagnamenta, 2017). These challenges are related to legal, technical, methodological, and financial issues that reduce the need for and limit the opportunities to conduct high quality evidence-generating studies (Pagnamenta, 2017; Gefen et al., 2018). The lack of collaboration with the industry (Harding et al., 2019) and competing demands of clinical research and clinical practice have also been highlighted (Pagnamenta, 2017).

Addressing evidence translation challenges is equally important. The meaning of evidence-based practice concept has shifted towards reliance on the methodological quality and epidemiological rigor of studies, which may hinder research translation, rather than foster a combined approach using best quality evidence, clinical judgement and including patient values (Pagnamenta, 2017). The need to improve the quality of studies in wound care remains (Weller and McNeil, 2010; Gethin et al., 2019). However, this is challenging within the real world clinical practice scenarios that include comorbid conditions and wound complexity (Harding et al., 2019) to ensure the chosen dressing ‘does no harm, (Gefen et al., 2018).

In making decisions regarding dressing product selection, clinicians should consider the patient’s comorbid conditions, wound assessment, dressing properties and cost (Dumville et al., 2013d; Dumville et al., 2015a; O'Meara et al., 2015) guided by evidence based wound management guidelines (Franks et al., 2016), which offer optimal and cost-effective care to help clinicians optimize healing outcomes in clinical practice (Cheng et al., 2018). The main contribution of this review is that it compiled the latest available evidence of effectiveness for the major groups of first-line dressings and provided a comprehensive discussion of the main disadvantages, contraindications, and precautions that can guide health professionals in decision making on dressing selection in addition to the clinical practice guideline recommendations. Succinct and clear summaries of first-line dressings with an organized approach can be used as a guide in clinical practice.

#### Limitations and Precautions

There are three main limitations of this review. Although it is a comprehensive scoping review, it should not be considered as systematic. First of all, our literature search was semisystematic, and we did not produce the PRISMA charts for the included groups of first-line dressings. Second, we aimed to focus on the data sources that provide the highest quality evidence, including RCTs and systematic reviews, and therefore, if these data sources were available, information on the effectiveness of first-line dressings based upon the findings from studies that provide lower level of evidence was excluded. Lower levels of evidence, however, were considered in the absence of higher quality evidence, including descriptive studies and pilot projects; for example, a pilot project on the use of hydrogel dressings for the management of Buruli ulcers (Velding et al., 2016). This study inclusion approach is considered appropriate, given the aim of this review was to compile the latest *available* evidence. In dressing selection, in the absence of high-quality evidence, an alternative mixed-method approach could be applied to obtain clinically valuable data and gain better understanding of a particular dressing (Pagnamenta, 2017). Thirdly, we were interested in the latest evidence of effectiveness of first-line dressings in wound management, and thus evidence produced before 2014 was not included unless there was no new evidence published. In this manuscript, specific products from various manufacturers have been provided as examples, and the list of dressing products should not be considered as exhaustive as there are many other products in the market. Finally, findings of this review should not be used as a separate guide to dressing selection. Health professionals are encouraged to refer to the wound-specific national clinical practice guideline recommendations to guide assessment, diagnosis, differential diagnosis, and management of wounds.

## Author Contributions

VT has conducted the literature search and drafted the manuscript with support and guidance from CW and GS. All authors critically reviewed and contributed to the individual parts of the manuscript, addressed reviewers’ comments, and approved the final version.

## Funding

This study is funded by the National Health and Medical Research Council (NHMRC) Translating Research into Practice (TRIP) Fellowship (NHMRC APP1132444) awarded to CW.

## Conflict of Interest

The authors declare that the research was conducted in the absence of any commercial or financial relationships that could be construed as a potential conflict of interest.

## Footnotes

1 We designed this table using multiple sources. These resources have been acknowledged in the reference list.
